# The Charity Organisation Society

**Published:** 1895-12-07

**Authors:** 


### THE CHARITY ORGANISATION SOCIETY.

Any society undertakes a considerable responsibility when it enters upon the field of journalism, for, in the absence of any disclaimer, the comments in its pages, however much they may be the work of a secretary's pen, must redound to the credit or the discredit of the whole body. In this connection we desire to draw attention to a statement published on pages 434 to 437 of the "Charity Organisation Review" for November, a publication issued at the offices of the Charity Organisation Society, but not bearing the name of any author or responsible editor. This statement, referring to a correspondence between Mr. Burdett and Mr. Loch, which has already been published, is transparently misleading, and altogether unworthy of the subject matter of the correspondence to which it relates. It contains much *suggestio falsi*, and we not only entirely decline to believe that the subscribers to the Charity Organisation Society will endorse the innuendoes conveyed in it, but we call upon them to publicly disclaim association with those who are responsible for the publication of such aspersions. We venture to think and to say that nothing so discreditable has appeared in the pages of the "Charity Organisation Review" since it has been published. Mr. Loch's attitude in regard to Canon Barnett, and now in regard to Mr. Burdett and the Inter-Denominational Committee of Friendly Workers, has caused great pain to some of the most able and powerful supporters of the C.O.S. His proceedings in both instances cannot fail to do an immensity of mischief to the society of which he is the secretary, and call for the strongest protest on the part of impartial people who value truth and fair dealing for their own sake. We hope our readers, or many of them, will take the trouble to procure copies of the "Charity Organisation Review" for November, that they may realise the dangers likely to be caused to any society by such unwarrantable behaviour as Mr. Loch has exhibited on these two occasions. Mr. Loch has done good service in many ways in the past; but, if he continues his present attitude, the influence of the C.O.S. must suffer considerably.

